# The relationship between central and peripheral oxytocin concentrations: a systematic review and meta-analysis protocol

**DOI:** 10.1186/s13643-016-0225-5

**Published:** 2016-03-31

**Authors:** Mathias Valstad, Gail A. Alvares, Ole A. Andreassen, Lars T. Westlye, Daniel S. Quintana

**Affiliations:** NORMENT, KG Jebsen Centre for Psychosis Research, Division of Mental Health and Addiction, University of Oslo, Oslo University Hospital, Oslo, Norway; Department of Psychology, University of Oslo, Oslo, Norway; Telethon Kids Institute, University of Western Australia, Perth, Australia

**Keywords:** Oxytocin, Plasma concentration, Cerebrospinal fluid concentration, ELISA, RIA, Systematic review, Meta-analysis, Protocol

## Abstract

**Background:**

Research examining the effects of oxytocin (OT) interventions on psychiatric, social-behavioral, and social-cognitive outcomes regularly collect peripheral levels of OT as markers of central bioavailability. Such inferences rest on the assumption that central and peripheral levels of OT are directly associated. However, conflicting evidence from coordinated sampling of central and peripheral OT question the validity of this assumption. The purpose of this meta-analysis is to evaluate the correlation between central and peripheral OT, as well as to account for potential heterogeneity in the literature.

**Methods/design:**

Studies that report coordinated sampling of central and peripheral OT in humans and animals will be identified. Research investigating concentrations in both basal states and after exogenous administration will be considered. PubMed and Embase databases will be searched, along with citation lists of retrieved articles. Peer-reviewed studies written in English published from 1971 onwards will be included in the meta-analysis. Data will be extracted from eligible studies for a random-effects meta-analysis. For each study, a summary effect size, heterogeneity, risk of bias, publication bias, and the effect of categorical and continuous moderator variables will be determined.

**Discussion:**

This systematic review and meta-analysis will identify and synthesize evidence to determine if there is an association between central and peripheral OT concentrations. If significant associations are observed, evidence would provide a rationale for future research to use peripheral measures as a proxy for central OT concentrations.

**Systematic review registration:**

PROSPERO CRD42015027864

**Electronic supplementary material:**

The online version of this article (doi:10.1186/s13643-016-0225-5) contains supplementary material, which is available to authorized users.

## Background

Oxytocin (OT) is a neuropeptide that is primarily synthesized in the paraventricular and supraoptical nuclei of the hypothalamus. OT plays a vital role in facilitating a range of physiological functions, such as labor induction and lactation [1]. However, recent research interest has focused more on its effects on social cognitive functions including emotion recognition, trust, and intersubjective selectivity [2–4]. Alongside these efforts determining the role of OT in typical behavior and cognition, research began to investigate the efficacy of this neuropeptide in the treatment of psychiatric disorders—such as autism spectrum disorder [5] and schizophrenia [6]—and the overall role of the OT system in psychopathology.

Three broad research approaches have been adopted to better understand the role of OT in cognition, behavior, and psychiatric illness: (i) the comparison of a psychological outcome measure or neurobiological effect after OT and placebo treatment; (ii) the comparison of basal OT concentrations between a pathological (e.g., psychiatric) and non-pathological control group; and (iii) the assessment of basal OT concentration covariance with various psychological or other biological phenomena. While central to the latter two methods in this list, peripheral concentrations of OT (e.g., blood plasma, saliva, urine) have been assessed within each of these research traditions, typically in an effort to approximate central OT concentrations and/or central bioavailability after OT administration. Using peripheral OT concentrations to index central concentrations is obviously appealing given the invasive methods required to collect centrally circulating fluids in humans, such as cerebrospinal fluid (CSF). However, the relationship between central and peripheral OT concentrations remains unclear. Although some animal research indicates that central release from the hypothalamus and peripheral release via the posterior pituitary is coordinated [7–9], other research does not support this [10, 11]. Research is also mixed in humans, with some results consistent with related levels of central and peripheral endogenous OT [12], while others report no significant associations [13]. In relation to exogenous OT, one human study found that peripheral and central levels were not related after intranasal administration; however, as the authors of the study acknowledge, the small sample size suggest caution in interpreting these results [14]. Together, it appears unclear if peripheral OT measures can be used as a proxy for central nervous system (CNS) concentrations and central OT bioavailability.

Meta-analysis enables statistical pooling of effect sizes and is a valuable tool for synthesizing results across studies and increasing power. In the proposed meta-analysis, we will examine studies in which both central and peripheral measures of OT have been sampled. This will enable greater understanding of the dynamics of neuropeptide release and distribution and help determine whether peripheral concentration is a reliable marker for central concentration.

As a primary outcome, an overall summary effect size for the relationship between central and peripheral concentrations of OT will be calculated from the effect sizes of all the individual studies included in the review. Since central and peripheral samples of OT have been obtained simultaneously in several species, the relevant population will not be limited to humans. This summary effect size will also generalize across experimental paradigms and interventions. Additional primary outcomes include summary effect sizes for the relationship between central and peripheral concentrations of OT assessments within species category and within experimental paradigm category (see below for details). The impact of other moderators will be treated as secondary outcomes.

## Methods/design

### Aims

The present study is a meta-analysis of available studies where coordinated central and peripheral measures of OT have been sampled. The aim is to examine the relationship between central and peripheral OT levels, as well as to provide a rationale for discrepancies between the results of independent studies. If a significant overall correlation is observed, then peripheral oxytocin concentrations may represent an index of central oxytocin levels. This protocol is registered with PROSPERO (CRD42015027864) and has been reported here according to the PRISMA-P [15] guidelines [see Additional file 1]. The procedures described in this protocol follow recent recommendations for the meta-analysis of correlational data [16].

### Search strategy

We will conduct a systematic literature search in three iterations to collect studies that simultaneously measure central and peripheral concentrations of OT. The specificity and sensitivity of different search criteria were examined in a pilot search. In the first iteration, searches will be performed in PubMed and Embase with the following combination of terms: (oxytocin) AND (concentration* OR level*) AND (plasma OR blood OR saliva* OR urin*) AND (central OR csf OR "cerebrospinal fluid"). Further limits for the search will be set in order to focus the search on full text articles that were published between January 1, 1971, and the date in which the search will be performed. In a second iteration, review articles will be consulted for relevant citations. Since any combination of terms is unlikely to exhibit perfect sensitivity to relevant articles, and review articles are unlikely to review a complete set of relevant articles, a third iteration will be performed in which reference lists within studies and citing articles of selected studies in the first and second iterations will be examined for remaining studies that include the critical measures.

### Inclusion and exclusion criteria

In this meta-analysis, we will include studies that meet the following criteria: (a) OT sampled in fluids with access to central (e.g., local extracellular fluid, cerebrospinal fluid) and peripheral (e.g., blood plasma, urine, saliva) regions of the body; (b) correlations between central and peripheral concentrations obtainable either through the article, the authors directly, statistical transformation of available data (e.g., Spearman to Pearson correlation), or “data scraping” from available scatterplots; (c) article published in a peer-reviewed journal between 1971 (discovery of ELISA) and June 2015; and (d) written in English.

### Moderators

Studies that provide simultaneous measures of central and peripheral OT concentrations are not only heterogeneous by the correlations they report, they also differ from each other on several potentially critical methodological aspects. These include species examined, participant characteristics, experimental paradigm, sampling type, biochemical analysis of OT concentration, as well as whether or not extraction of neuropeptide from sampled substance was performed. The following potential moderator variables will be examined a priori in this meta-analysis in order to account for heterogeneity in the literature.

1. *Participants and species.* Participants include clinical and non-clinical human populations. Animal studies will also be included to collect a broader dataset of studies assessing peripheral and central OT concentrations, with species encompassing non-human primates and other mammals. It is possible that studies in non-human animals may yield results differing from those in human studies given differences in physiology to humans. For example, the olfactory epithelium covers as much as 50 % of the rodent nasal cavity [17] whereas the proportion in humans is only 3–15 % [18, 19]. As intranasal administration of OT appears to depend on uptake through the olfactory epithelium for direct entry into the brain [20], such differences in physiology moderate effect sizes. Furthermore, within human studies, we will examine whether effect sizes are moderated by (a) gender of participants, (b) presence or absence of physical illness, and (c) presence or absence of psychiatric diseases.
2. *Experimental paradigms.* These range from the examination of baseline levels of central and peripheral OT [13, 21], and levels following some environmental stimulus such as light [22], and stressors [7, 23], to examinations of concentrations after manipulation of OT levels by osmotic stimulation [9], drug administration [24–26], or the administration of intranasal [14, 27] or intraperitoneal [27] OT.
  Since the pharmacokinetics of OT seem to exhibit quite complex, time-dependent patterns [9], there is a possibility that differences in experimental designs account for differences in correlations between studies. For this moderator, (a) baseline samples, (b) samples after exogenous intranasal OT administration, (c) samples after intravenous or intraperitoneal OT administration, (d) samples after intracerebral OT administration, and (e) samples after other experimental manipulations (e.g., osmotic stimulation) will be treated as levels within the experimental paradigms moderator variable. In studies that report both baseline correlation and correlations after manipulation, the baseline sample will be assigned to the baseline level in the moderator analysis, while experimental samples will be assigned to experimental levels.
3. *Sampling types.* These include CSF concentrations and concentrations in central extracellular fluids, and measures of concentrations in peripheral blood, urine, and saliva. A number of discrepancies have been seen in reports of OT concentration measures. For example, urine samples have been reported to give OT concentration estimates at a million times above usual values for plasma samples [28]. Furthermore, in a study on OT levels in parents interacting with their infants, Feldman et al. [29] found no correlation between urine concentrations of OT and concentrations in blood plasma or saliva. In studies that administer OT intranasally, large amounts of the neuropeptide may be drawn through the floor of the nasal vault, missing nose-to-brain targets in the upper posterior regions, down to the oral mucosa and gastrointestinal tract. In these cases, it is difficult to distinguish between exogenous OT that has dripped down into the oral cavity and salivary OT that represent systematically circulating levels. Indeed, saliva OT has been examined for its validity as a biomarker, with negative results reported [30].
4. *Method for biochemical analysis of OT concentration*. Two main methods for biochemical detection of sampled substance are reported in the literature: enzyme immunoassay (EIA) and radioimmunoassay (RIA). In a validation procedure directed at these methods, Christensen et al. [31] found that RIA was more sensitive to low concentrations of OT and that concentration estimates provided by RIA corresponded more closely to estimates by highly specific two-dimensional liquid chromatography-tandem mass spectrometry assay [32, 33]. Thus, studies that employ EIA might exhibit increased amounts of noise and accordingly weakened correlations between central and peripheral oxytocin. Another related concern regarding biochemical analysis of OT concentration is whether or not neuropeptides are extracted from the sampled substance prior to EIA or RIA.
  Extraction of neuropeptides from the sampled substance is not universal among studies [31, 34]. Measures taken from raw substance are more susceptible to cross-sensitivity, resulting in values that reflect variability of unrelated molecules in addition to the true level of oxytocin [34, 35]. Szeto et al. [36] found estimates of OT concentrations using EIA to be two orders of magnitude higher in non-extracted plasma as compared to extracted, additionally finding no significant association between OT level estimates from extracted and non-extracted plasma. Thus, there is a possibility that studies where substances are extracted prior to immunoassay provide more accurate correlations between central and peripheral oxytocin if collected simultaneously. In addition, Szeto et al. [36] found that while EIA estimates increased a 100-fold in extracted plasma as compared to non-extracted, extraction did not increase sensitivity of biochemical analysis using RIA. There seems, in other words, to be an interaction effect of analysis method and extraction on OT concentration estimates. Accordingly, there might be an interaction effect of analysis method and extraction on estimates of correlations between central and peripheral OT concentrations. Therefore, in this meta-analysis, the moderator biochemical analysis method contains two variables with two levels each (RIA vs EIA, and extraction prior to immunoassay or not), and we will examine the main effects of both variables as well as interaction effects.
5. *Age*. CSF concentrations of OT seem to be related to age in macaque monkeys, decreasing with age in infants while increasing with age in mothers [37]. Changes in the oxytocin system with age [38, 39] may also involve the extent of coordinated release or even the degree to which OT may penetrate the blood brain barrier.
6. *Risk of bias*. A risk of bias tool has been developed to quantify potential biases in effect sizes related to study quality (see below).
7. *Year of publication*. Early, more preliminary studies, may potentially exhibit different effect sizes, so year of publication is included as an additional moderator to assess bias.
8. *Level of sample coordination*. In some studies, central and peripheral samples are collected simultaneously, whereas in others, there is a brief interval between central and peripheral samples [21]. In this meta-analysis, levels of sample coordination will be coded as simultaneous or non-simultaneous.

### Quality assessment (risk of bias)

A custom tool will be developed to assess risk of bias via a systematic procedure for determining the quality of eligible studies [see Additional file 2]. The risk of bias tool is adapted from a previously used tool [40]. Using this tool, studies will be given a score of potential bias by two raters. This tool, in combination with publication bias (described below), will be used to assess the quality of overall evidence. The evidence will be categorized as high (no evidence of publication bias and mean quality scores are ≥80 %), moderate (little-to-no publication bias and quality scores are ≥50 and <80 %), low (evidence of publication bias and quality score ≥50 %), or very low (evidence of publication bias and quality score <50 %).

### Data extraction and management

A summary effect size for all studies will be computed with effect sizes (correlations transformed to Fisher’s *z*) and sample sizes from individual studies as input. Effect sizes will be extracted using one out of four possible procedures, depending on whether and in what form effect sizes are reported in a study. First, if effect sizes are reported, these will be transformed to Fisher’s *z*. Secondly, if effect sizes are not reported but individual results are reported numerically, *r* will be calculated and transformed to *z*. Thirdly, in studies published after 2000 (as 15 years is a common time frame for the retention of clinical data) that do not report effect sizes or individual results in numerical form, authors will be contacted and asked to provide the relevant information. Fourth, in studies either published before 2000 or where authors do not respond to our request, and where all individual results are reported in scatterplot graphs, a plot digitizer (http://arohatgi.info/WebPlotDigitizer) will be used for conversion into numerical values, from which *r* will be calculated and transformed to *z*. Studies that do not fall into one of these four categories do not provide the data that is necessary for our analysis and will not be included in the meta-analysis.

Two independent reviewers will extract data from all eligible studies using the data extraction form [see Additional file 3]. This form includes (a) general information on studies including authors, title, number of effect sizes, sample size, and effect sizes; (b) information about the participants including species, gender, age, and physical and mental health status; (c) information about the level the study is on other moderator variables, including study type, biochemical analysis, and neuropeptide extraction; as well as (d) information concerning study quality including publication year and the risk of bias measures as defined by the custom risk of bias tool. Any disagreements will be adjudicated by author DSQ.

The data will be entered into an excel version of the data extraction form, with one excel sheet for each reviewer. For each study, the extracted data will be entered in a separate tab of the data extraction excel sheet. After the data extraction phase of this meta-study, the data will be saved as .csv files and treated as data frames in R for analysis. Data from studies initially selected based on title and abstract and articles included in the review will be documented. Reasons for the exclusion of retrieved articles will also be recorded for eventual documentation in a study search and data extraction flow diagram.

## Statistical analysis

Analysis will be performed with R statistical software version 3.1.1. [41], using the metafor [42] and robumeta [43] packages. All effect sizes will be converted to Fisher’s *z* prior to analysis, and back to Pearson’s *r* after analysis. Operations needed for the analysis include the following:

### Summary effect size

Since the designs of individual studies vary substantially (e.g., differing species, biochemical methods), real effect sizes of individual studies should vary accordingly. Thus, a random effects model is appropriate in the computation of summary effect size [44]. Summary effect size as well as confidence interval and statistical significance of effect size will be computed in R. In the case of studies reporting more than one correlation, Robust variance estimation will be used with adjusted estimators for small samples (*n* < 40), if necessary.

### Sample heterogeneity

The *Q*-statistic is the sum of the products of weights and squared differences between study effect sizes and summary effect sizes. As such, it is an expression of the heterogeneity of effect sizes in the sample. A significant *Q*-statistic is indicative of significantly different effect sizes between the studies included in the meta-analysis. In this meta-analysis, we follow the convention of an alpha level of .05 for the *Q*-statistic. *I*^2^ reflects the proportion of total variance that is attributable to real between studies variance. The *I*^2^ statistic is useful for determining whether the amount of real between studies effect size variance is relatively higher than chance variability. The *Q*-statistic, the significance of the *Q*-statistic, and *I*^2^ will be computed and reported.

### Categorical moderator analysis

A random effects model with separate estimates of between studies variance will be applied for all categorical moderator variables (species, paradigm, sampling type, biochemical method, and extraction). Summary mean effects are calculated for each subgroup, as described in the previous section for summary effect sizes. *T*^2^ is calculated separately for each subgroup. Means are compared using a two-tailed *z*-test in order to determine the probability for the difference of observed means given equality of true means, assuming normal distribution. A *p* value below .05 will be seen as indicative of moderating properties of the variable in question.

### Continuous moderator analysis

For the continuous moderator variables (age, risk of bias, and year of publication), meta-regression will be performed to estimate an unstandardized regression coefficient along with the coefficient’s significance level.

### Publication bias

Publication bias will be assessed using the regression test for funnel plot asymmetry outlined in Egger et al. [45]. If there is evidence of publication bias, we will use the Trim and Fill method, which provides a reasonable approximation of “missing” effect sizes [46]. With this method, the asymmetric right side of the funnel (which is the side expected to be effected by publication bias in the present meta-analysis) is “trimmed”. The remaining symmetric effect sizes are then used to re-estimate the center of the funnel, after which the trimmed effect sizes and their missing counterpart effect sizes on the left side of the funnel are “filled”. An estimate of the summary effect size and variance will then be calculated on the filled funnel plot as a form of sensitivity analysis to examine the potential impact of missing studies.

## Discussion

There is increasing research on the use of peripheral OT concentrations to determine relationships to a range of psychological and neurobiological phenomena. Moreover, levels of peripheral OT are also used to determine the bioavailability of OT after intranasal administration. These measures are often used as a proxy for central levels of OT but it is unknown if these two measures are in fact related, with mixed results reported in the literature across species and experimental designs. Recent debate has also highlighted the need for a more thorough characterization of OT mechanisms [47, 48] which includes measures of central and peripheral OT bioavailability.

This will be the first systematic review and meta-analysis, to the best of our knowledge, to investigate this association. An up-to-date synthesis of the available data will help determine if future research can use peripheral levels of OT as a measure of central activity or if the collection of CSF concentration is necessary to make these conclusions. Alternatively, the proposed meta-analysis may indicate that central and peripheral OT concentrations are only related under certain conditions, such as after exogenous OT administration. Predetermined measures of publication bias and study quality will be used to describe the confidence in the resulting body of evidence.

## Additional files

Additional file 1: PRISMA-P (Preferred Reporting Items for Systematic Review and Meta-Analysis Protocols) 2015 checklist. Recommended items to address in a systematic review protocol. (PDF 165 kb) Additional file 2: Risk of bias tool. Criteria for objective assessment of risk of bias for individual studies included in the meta-analysis. (PDF 37 kb) Additional file 3: Data extraction form. Record of pertinent study characteristics for each included study. (PDF 37 kb)

## Abbreviations

CNS: central nervous system
CSF: cerebrospinal fluid
EIA: enzyme Immunoassay
OT: oxytocin
RIA: radioimmunoassay
