# Supplementary material for: The relationship between central and peripheral oxytocin concentrations: a systematic review and meta-analysis protocol
Source: Syst Rev. 2016 Mar 31;5:49. doi: 10.1186/s13643-016-0225-5 (PMC4818503; doi:10.1186/s13643-016-0225-5)
Supplement: Additional file 2: — Risk of bias tool. Criteria for objective assessment of risk of bias for individual studies included in the meta-analysis. (PDF 37 kb) [file 13643_2016_225_MOESM2_ESM.pdf]

Additional file 2: Risk of bias tool. Criteria for objective assessment of risk of bias for individual studies included in the meta-analysis.

Criteria	Score
**Category 1: Sample characteristics**	
Human population	
1. Presence (or absence) of study-relevant diagnosis established by trained assessor [1p], according to standardized international criteria [1p].	/2
2. Reported comorbid diseases [1p] and medication use [1p].	/2
Non-human population	
3. Specified species [1p], source of individual animals stated [1p].	/2
**Category 2: Internal Validity**	
4. Potential confounding variables controlled for [2p], or accounted for [1p].	/2
5. All [2p] or most [1p] outcome measures have been validated.	/2
**Category 3: Methodology and reporting**	
6. Method of analysis stated for central [1p] and peripheral [1p] measure.	/2
7. Outcome data is complete, or incompleteness is accounted for [2p]	/2
8. Method of extraction described [1p].	/1
**Category 4: Paradigm-specific criteria**	
9. IF IN-OT: Proper instructions to participants for administration of OT [1p], measure of nasal cavity health [1p].	/2

For a single study, a raw score of 11-15 is obtainable in principle. For each study, the risk of bias is expressed as a ratio of the actual raw score to its obtainable raw score. Abbreviations: OT = oxytocin, IN-OT = intranasal oxytocin.
