# Supplementary material for: The relationship between central and peripheral oxytocin concentrations: a systematic review and meta-analysis protocol
Source: Syst Rev. 2016 Mar 31;5:49. doi: 10.1186/s13643-016-0225-5 (PMC4818503; doi:10.1186/s13643-016-0225-5)
Supplement: Additional file 3: — Data extraction form. Record of pertinent study characteristics for each included study. (PDF 37 kb) [file 13643_2016_225_MOESM3_ESM.pdf]

Additional file 3: Data extraction form for individual studies included in meta-analysis of correlations between central and peripheral levels of oxytocin.

**Categories**	**Values**
**A Identification of study**	
1. Study ID	
2. Authors	
3. Title	
4. Year of publication	
**B Primary values**	
1. Number of correlations	
2. Sample size	
3. Effect size	
4. Number of samples	
**C Population**	
1. Species	
2. Gender	
3. Mental health status	
4. Somatic health status	
5. Age	
**D Moderators**	
1. Study type	
2. Sampling location	
3. Biochemical analysis	
4. Extraction	
5. Level of coordination	
**E Other**	
1. Risk of bias	

A1: Assign for each study included. **B1**: Specify number of reported correlations. **B2**: For each correlation, specify sample size. **B3**: For each correlation, specify effect size and entity of correlaton. **B4**: For each correlation, specify included number of time-points for coordinated samples. **C3**: Specify mental health status of participants. **C4**: Specify somatic health status of participants. **C5**: Specify age of participants in range and/or mean and standard deviation. **D1**: Specify whether concentrations are sampled at a) baseline level, b) after exogenous OT administration, or c) after other experimental manipulations. **D2:** Specify the locations for central and peripheral samples. **D3**: Specify method for biochemical analysis of OT concentration. **D4**: Specify whether OT was extracted from the sampled substance. **D5**: Specify whether central and peripheral concentrations were sampled a) simultaneously (time interval < 2 mins), b) non-simultaneously, or c) not reported. **E1**: Report risk of bias expressed as a ratio of raw score to obtainable raw score for the study (see risk of bias form).
